# Ventricular Arrhythmias in First Acute Myocardial Infarction: Epidemiology, Mechanisms, and Interventions in Large Animal Models

**DOI:** 10.3389/fcvm.2019.00158

**Published:** 2019-11-05

**Authors:** Stefan Michael Sattler, Lasse Skibsbye, Dominik Linz, Anniek Frederike Lubberding, Jacob Tfelt-Hansen, Thomas Jespersen

**Affiliations:** ^1^Department of Cardiology, Heart Centre, Copenhagen University Hospital, Copenhagen, Denmark; ^2^Medical Department I, University Hospital Grosshadern, LMU Munich, Munich, Germany; ^3^Department of Exploratory Toxicology, H. Lundbeck A/S, Copenhagen, Denmark; ^4^Medical Department III, Universitätsklinikum des Saarlandes, Homburg, Germany; ^5^Centre for Heart Rhythm Disorders, South Australian Health and Medical Research Institute, Royal Adelaide Hospital, University of Adelaide, Adelaide, SA, Australia; ^6^Department of Biomedical Sciences, Faculty of Health and Medical Sciences, University of Copenhagen, Copenhagen, Denmark; ^7^Department of Forensic Medicine, Faculty of Medical Sciences, University of Copenhagen, Copenhagen, Denmark

**Keywords:** acute myocardial infarction, ventricular arrhythmia, animal models, anti-arrhythmia agents, sudden cardiac death, STEMI, ischemia, ventricular fibrillation

## Abstract

Ventricular arrhythmia and subsequent sudden cardiac death (SCD) due to acute myocardial infarction (AMI) is one of the most frequent causes of death in humans. Lethal ventricular arrhythmias like ventricular fibrillation (VF) prior to hospitalization have been reported to occur in more than 10% of all AMI cases and survival in these patients is poor. Identification of risk factors and mechanisms for VF following AMI as well as implementing new risk stratification models and therapeutic approaches is therefore an important step to reduce mortality in people with high cardiovascular risk. Studying spontaneous VF following AMI in humans is challenging as it often occurs unexpectedly in a low risk subgroup. Large animal models of AMI can help to bridge this knowledge gap and are utilized to investigate occurrence of arrhythmias, involved mechanisms and therapeutic options. Comparable anatomy and physiology allow for this translational approach. Through experimental focus, using state-of-the-art technologies, including refined electrical mapping equipment and novel pharmacological investigations, valuable insights into arrhythmia mechanisms and possible interventions for arrhythmia-induced SCD during the early phase of AMI are now beginning to emerge. This review describes large experimental animal models of AMI with focus on first AMI-associated ventricular arrhythmias. In this context, epidemiology of first AMI, arrhythmogenic mechanisms and various potential therapeutic pharmacological targets will be discussed.

## Introduction

Despite a decrease in overall cardiovascular mortality over the past decades, ~17 million deaths a year occur worldwide as a result of cardiovascular disease (1) and ~50% of these are reported to be sudden cardiac deaths (SCD) (2). In the elderly, constituting the major age group at risk, SCD is often associated with chronic degenerative diseases, such as coronary artery disease (with acute myocardial infarction (AMI) as its ultimate consequence), valvular diseases, and heart failure.

In the early stages of AMI ventricular fibrillation (VF) is a frequent cause of SCD, which often occurs within minutes after onset of clinical symptoms, often even before the patient has established contact to medical health care systems. As out of hospital VF following AMI is sudden and unexpected, only sparse information concerning risk factors and protective mechanisms are available from human studies.

Hence, to understand the mechanisms of ventricular arrhythmias developing during AMI and to discover new treatment modalities for these patients, investigations through translational experimental models, mimicking the clinical situation, are needed. While numerous experimental models of myocardial infarction exist, most of them focus on mechanisms and on options to prevent manifestation and expansion of infarction and scar formation in order to improve tissue survival in post-AMI patients. Only a relatively limited number of studies focus on the acute arrhythmogenic risk of AMI. This in spite of the fact, that there is a critical need for fast acting acute antiarrhythmic treatments in AMI patients. Treatment that is safe to use on site or in an ambulance without severe negative hemodynamic or proarrhythmic adverse effects. Hence, there is an unmet medical need for such therapeutic options, which calls for investigations of novel pharmacological targets in clinical and preclinical antiarrhythmic research.

This review will present the published literature on experimental large animal models of AMI with focus on translatability to the epidemiology of first AMI-associated ventricular arrhythmias. Various therapeutic pharmacological targets will be discussed in this context. Along with this, a brief overview of the involved arrhythmogenic mechanisms leading to VF during AMI is given.

## Acute Myocardial Infarction and Sudden Cardiac Death

**Figure 1:**
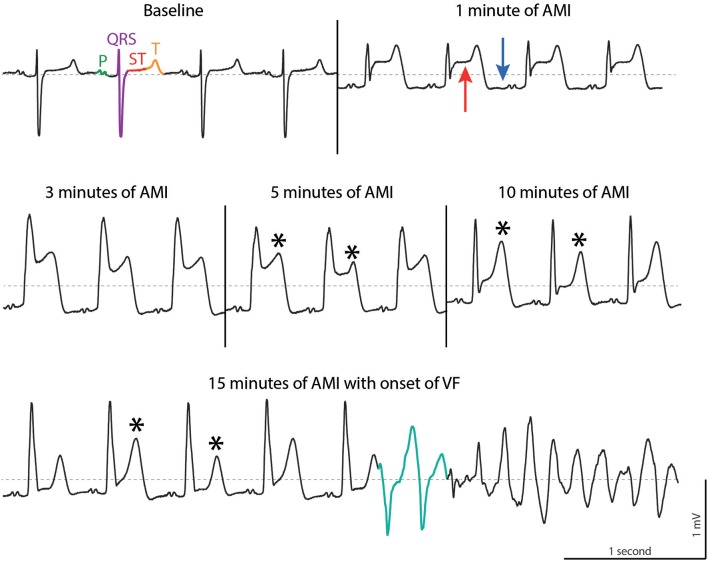
ECG tracings during acute myocardial infarction (AMI) in a porcine model. At baseline, sinus rhythm is present with P wave (green), QRS complex (purple), ST segment (red) and T wave (orange). AMI leads to elevation of the ST segment (red arrow), depression of the TQ segment (blue arrow) followed by T wave alternans (asterisk, note the alternating morphologies). In this example, ventricular fibrillation is triggered by two short coupled ectopic beats (turquoise). Dotted line, 0 mV. Reproduced with permission from Sattler (7).

Ischemia-related VF is by far the leading cause of sudden cardiac arrest and the most frequent occurring arrhythmia in SCD (2–5). In AMI, occlusion of a coronary artery induces an imbalance between oxygen supply and demand leading to ischemia-induced cell death of cardiac myocytes. Transmural AMI, seen as ST-elevation myocardial infarction (STEMI) in the electrocardiogram (ECG) is present in 25–40% of all AMI cases (6). AMI rapidly changes electrophysiological properties of the ventricular myocardium and, in a significant proportion of patients, promote electrical disturbances in conduction and repolarization, leading to ventricular tachycardia (VT) or VF and subsequently cardiac arrest (Figure 1) (6). Due to development of myocardial scar tissue and heart failure, ventricular arrhythmias and subsequent SCD can still occur after transition from acute to chronic myocardial infarction.

By nature, it is difficult to establish the cause of death to be VF caused by AMI when it happens out of hospital. The numbers in the following studies likely underestimate the true burden of VF during first AMI because individuals experiencing SCD are not reported. Many patients with ischemia-induced VF will be found dead, or, after being resuscitated, die or survive with or without neurological sequelae (8, 9). Even if medical contact is established, only 4.3–19.4% of the patients experiencing out-of-hospital cardiac arrest (OHCA) survive the first 30 days (8). In patients with STEMI, 30-days survival decreases from 94% in patients without VF to 79% in those with VF (10). In our own Danish nationwide prospective case-control study, the GEVAMI (GEnetic causes of Ventricular Arrhythmias in patients with first ST-elevation Myocardial Infarction) study, we enrolled 660 consecutive STEMI patients and found 11.6% to develop VF before primary PCI (11). The French e-MUST (Evaluation en Médecine d'Urgence des Stratégies Thérapeutiques des infarctus du myocarde) study reported that 5.6% of patients with STEMI experienced VF witnessed by emergency medical service (EMS) on site. Of these patients, 9.1% died out-of-hospital and a further 29% in-hospital (12).

### Myocardial Infarction and VF Risk

The assessment of risk factors of VF due to AMI is non-trivial: Studies must be designed to distinguish between risk factors for AMI without VF and those for AMI with VF. Further, patients suffering from SCD before AMI is diagnosed are often not included in such studies. Finally, these events occur in low risk subgroups without any known risk factors, like structural heart disease (4). Possible risk factors for VF can either be present before AMI takes place or can be directly associated with specific factors of the AMI itself. The former are determined by heritability, comorbidity, medication, lifestyle, and gender, while the latter, mostly observed as changes in the ECG, includes infarct size and location of coronary obstruction, as well as individual ventricular and coronary anatomy (11, 13).

#### Pre-AMI Risk Factors

The Dutch AGNES (Arrhythmia Genetics in the Netherlands) study was the first designed to differentiate between risk factors for AMI in general and AMI complicated by VF (13). This study compared first AMI patients who developed VF with AMI patients without VF. Those with VF had a family history of SCD, a lower body mass index, and a lower prevalence of hypercholesterolemia and diabetes compared to controls.

The GEVAMI study compared first STEMI with and without VF and was able to identify several independent risk factors for VF including age below 60 years, family history of SCD, absence of pre-infarction angina, use of statins, history of atrial fibrillation (AF) and alcohol intake (> 7 units per week) (11). Whereas, alcohol has favorable effects on the development of thrombosis and atherosclerosis and lowers the risk of AMI (14), a u-shaped relationship exists in SCD. A moderate consumption of alcohol (2–6 units a week) reduces the risk for SCD (15, 16), while higher alcohol intake increases the risk (17).

Several studies support the theory that a genetic predisposition to SCD and VF exists in the setting of AMI (13, 18). A family history of SCD among siblings or parents increased the risk of VF (odds ratio of 1.6–3.2) (11, 13, 19). However, little is known about the specific genetic factors affecting VF during AMI. A genome wide association study (GWAS) in the AGNES study cohort revealed a strong association between VF during AMI and a gene locus near the CXADR gene, that encodes for the coxsackie- and adenovirus receptor (20). Smaller studies point at genetic variations in the SCN5A gene, coding for the cardiac sodium channel Na_v_1.5, as a predisposing factor for the development of VF upon AMI (21). Epidemiology and genetics of VF in AMI have been reviewed recently by Glinge et al. (22).

Another important confounder to VF in AMI is AF (11, 13). Data from the FAST-MI (French ST-elevation and non-ST-elevation Myocardial Infarction) 2005 registry point at AF observed on the initial ECG obtained during STEMI to be an independent risk factor for early VF (odds ratio 2.5) (23). Increased heart rate in AF can shorten ventricular refractoriness creating a substrate for VF (24). Additionally, this proarrhythmic substrate can be aggravated by hemodynamic changes that can decrease parasympathetic and increase sympathetic tone in AF (25).

#### AMI Risk Factors

Properties of AMI itself including infarct size and location can alter the incidence of VF. Electrocardiograms obtained in the early phase of AMI are therefore not only important for diagnosing AMI, but also in early risk stratification. Patients presenting with high ST elevation score (11, 26), short RR intervals (27) or prolonged PR and QRS intervals (26) in their ECGs carry a higher risk of VF. Additionally, patients with ST-segment elevation accompanied with distortion of the terminal QRS complex had increased ischemia burden, faster progression of necrosis (28), and a higher risk of VF (26).

Incidence of VF is also influenced by the affected artery or arteries. A Belgian case-control study, investigating cardiac arrest, found an increased risk of out-of-hospital VF in patients with acute occlusion on the left coronary artery (left anterior descending (LAD) or left circumflex (LCx) artery) compared to the right coronary artery. However, the exact occlusion site (proximal or distal part) within the left coronary artery did not affect VF burden (29). Similar results were found in the Dutch AGNES study (13). This is somehow conflicting as proximal occlusion sites result in larger areas of myocardium at risk and have been shown to be a risk factor in animal models (30, 31). Reasons for these discrepancies could be manifold including time to reopening of the occluded vessel, residual flow in the coronary artery, or the limited number of patients.

## Cardiac Electrophysiology and Arrhythmogenesis

AMI causes a variety of electrophysiological changes generating triggering factors and a proarrhythmic substrate that can induce and sustain VF. In this section we give a brief introduction to important concepts on cardiac electrophysiology and arrhythmogenesis during ischemia. For a more in depth review we refer to the reviews by Janse and Wit (32) and Carmeliet (33).

**Figure 2:**
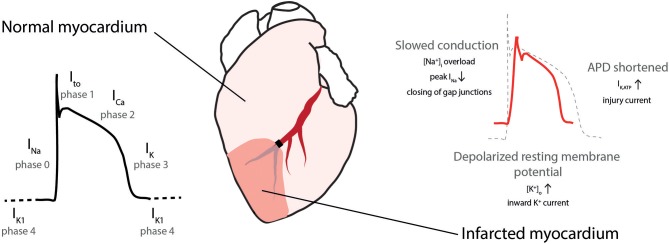
Cardiac action potential in normal and ischemic myocardium. **(Left)** Action potential with depolarizing sodium (I_Na_), transient outward potassium (I_to_), calcium (I_Ca_), and repolarizing potassium (I_K_) currents. **(Right)** During ischemia conduction is slowed, the resting membrane potential depolarized and action potential duration (APD) is shortened. [Na^+^]_i_, intracellular sodium concentration, [K^+^]_o_ extracellular potassium concentration, I_K,ATP_, ATP-sensitive potassium current. Modified with permission after Sattler (7).

The cardiac action potential (AP) is formed following fine-tuned temporal and spatial openings of depolarizing sodium (Na^+^) and calcium (Ca^2+^) and repolarizing potassium (K^+^) channels (Figure 2, left) (34). AMI alters the metabolic state (mainly via acidosis and ATP depletion) and causes electrical changes in both the ischemic area and the border zone between the ischemic and non-ischemic area. Numerous factors have been proposed to support the generation of arrhythmias: The resting membrane potential is depolarized, conduction velocity in the ventricles slows down, and AP duration shortens by alterations in the K^+^ and Cl^−^ currents during AMI (Figure 2, right) (32, 35).

**Figure 3:**
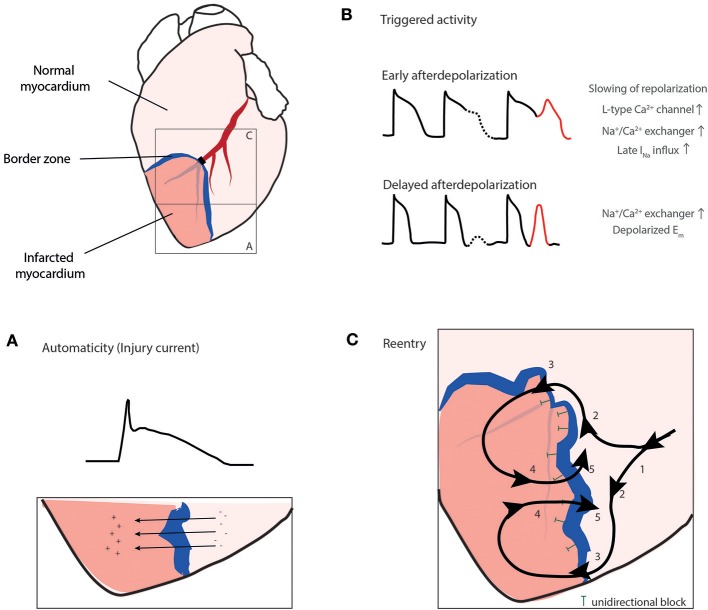
The three mechanisms, automaticity **(A)**, triggered activity **(B)**, and reentry **(C)** can play a role in arrhythmogenesis during ischemia. **(A)** Injury current across the border zone leading to ST elevation in the electrocardiogram, **(B)** Triggered activity mainly caused by Ca^2+^ overload in cardiomyocytes or Purkinje fibers. **(C)** Reentry. Electrical activation wave front (1) is deflected at the border zone due to unidirectional block (T) into two wave fronts (2), eventually passing the border zone (3) and exciting the infarct zone (4) and finally passing the unidirectional block re-exciting the area in front of the block (5). I_to_, transient outward potassium current; [K^+^]_o_, extracellular potassium concentration; [Na^+^]_i_, intracellular sodium concentration. Reproduced with permission from Sattler (7).

During ischemia three underlying mechanisms, automaticity, triggered activity, and reentry can be involved in inducing or sustaining arrhythmias (premature beats, VT and VF; Figure 3) (33). Automaticity is caused by myocardial stretch, or short-circuit and injury currents in the border zone leading to spontaneous depolarizations outside the sinus node. Triggered activity is caused by Ca^2+^ overload in Purkinje fibers or cardiomyocytes. The Purkinje system is thought to play a role in the transition of a stable reentrant VT into VF by rapid and irregular conduction of reentrant waves into non-ischemic tissue (36). Finally, reentry is caused by wave fronts following a pathway around an area of unidirectional block and reexcitement of previously activated cardiomyocytes instead of dying out. Reentry occurs in the presence of a functional disturbance in impulse conduction and heterogeneity in conduction as present during AMI (32).

Early studies on VF used rather simplified concepts to explain VF initiation, including a single stable reentry circuit traveling along an anatomical obstacle or the leading circle theory in which a wave front travels around a functional obstacle that is constantly kept depolarized (37). Rotors, a form of functional reentry where the curved wave front and wave tail meet each other in an excitable singularity, have emerged as an explanatory theory for VF in the last decades: A propagating wave front with a curvature *R* (source) travels along an obstacle (for example an unidirectional block) and is about to enter excitable but non-excited tissue (sink). The curvature *R* is dependent on electrophysiological properties like conduction velocity. At the edge of the obstacle, the curvature *R* determines whether the wave will detach from the obstacle or follow it. If R is smaller than the critical radius of the obstacle, the wave will eventually detach, curl and rotate around its broken tip (37). In VF initiation and maintenance, a small number of driving rotors (mother rotor) and multiple wavelets have been detected in humans with VF and are thought to be important (38).

Autonomic imbalance, characterized by increased sympathetic activation and parasympathetic withdrawal, may also contribute to the early manifestation of ventricular arrhythmia in the acute phase of AMI as well as following AMI (39, 40).

### Arrhythmias During Ischemia

Changes in ionic currents and the development of an arrhythmic substrate during early AMI have a defined time course and ventricular arrhythmias (premature beats, VT and VF) occur in two distinct phases, 1a and 1b (32, 41).

Phase 1a takes place between 2 and 10 min after AMI and is induced by focal activation due to injury currents in a substrate of profound slowing in conduction velocity and delayed activation due to decreased resting membrane potential (41). Reentry is the predominant mechanism during this phase, but triggered activity can also be involved in the generation of premature beats (32, 42).

Phase 1b occurs 18–30 min after coronary occlusion. Arrhythmias are likely related to mechanical stretch in the border zone, increased catecholamine levels and cellular uncoupling (43–45).

Overall incidence of arrhythmias is higher during phase 1a while VF occurs more often during phase 1b (46). Data in humans on the importance of phase 1a and 1b on SCD are scarce, since ECG recordings in patients are rare, while animal models suggest larger mortality in phase 1b than in phase 1a (47–49).

## Animal Models of Ventricular Arrhythmias in Acute Ischemia

Animal models are essential in understanding the mechanisms of identified risk factors on arrhythmogenesis and subsequent SCD. Mimicking the exact pathophysiological conditions in experimental models translatable to AMI in humans can be challenging. Ideally the experimentally-induced AMI results in the same pathophysiological changes that would be expected in a diseased human, however, experiments in smaller animals have limitations in translatability to humans. This is mainly due to differences in the dimensions of the ventricles, the heart and the cardiovascular system as a whole, the morphology and duration of the AP, as well as differences in hemodynamic and cardiac electrophysiological responses to ventricular ischemia (50). In contrast, large animal models provide a better opportunity to study the pathophysiology and pathogenesis of AMI and associated arrhythmias (32) and allow the use of established invasive techniques also applied in the clinic, such as coronary angiograms, multisite electrogram recordings, stimulation protocols, electrode arrays, or whole electrode mapping-socks covering the entire heart (51, 52).

Besides the applied species, the occurrence of studied ventricular arrhythmias depends on a number of factors, including anesthetic regimen, mode of coronary occlusion, occlusion site and size of the ischemic area, coronary collaterals, and the regulation of the autonomic nervous system.

### Anesthesia

The vast majority of animal models developed for understanding AMI-induced ventricular arrhythmias have concerned fully anesthetized animals, although, notable differences can be observed between conscious and anesthetized animals (53). Numerous different anesthetic drug-regimens have been used, as reviewed by Hamlin (54). Anesthetics have different abilities to sustain an adequate anesthesia but often come with unwanted effects on susceptibility of ventricular arrhythmias, sympathetic and parasympathetic regulation, and on the cardiovascular system itself.

Briefly, many studies investigating arrhythmias in animals have used barbiturates. These drugs have strong parasympatholytic effects, reducing transmural dispersion of ventricular repolarization (55), leading to a less frequent occurrence of ventricular arrhythmias (56, 57). A comparison of the volatile anesthetics isoflurane and sevoflurane in pigs revealed a higher VF and mortality rate in the isoflurane group (58). Propofol is an anesthetic drug acting mainly on the gamma-aminobutyric acid (GABA) receptor and is widely used nowadays as it is easy to dose. It comes with notable effects on hemodynamics, including reduced cardiac output, venous return, systemic pressure, and vascular resistance (59). In studies that investigate autonomic regulation of the cardiovascular system and effects of cardiovascular drugs the use of morphine and alpha chloralose (60, 61) or urethane and alpha chloralose (62) might be considered. The use of morphine and alpha chloralose has lower effects on the cardiovascular- and autonomic nervous system compared to other regimens and create a state comparable to sleep (63). Thus, when the focus is placed on assessing risk predictors and effects of antiarrhythmic treatments, the effects of the used anesthetic regimen must be taken into account.

### Mode of Infarct Induction

**Figure 4:**
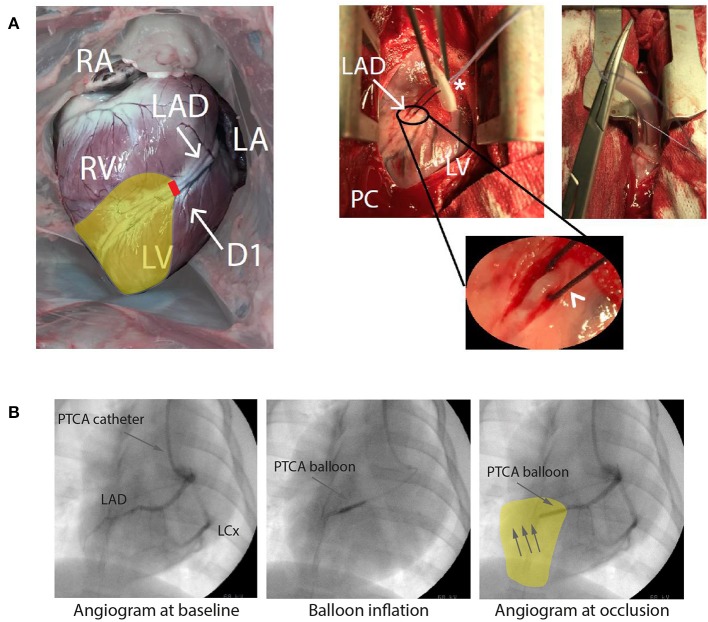
**(A)** Open chest approach. Visualization of a heart from a Danish Landrace pig after mid-thoracotomy in anterior-posterior view. Right (RA) and left atrium (LA), right (RV), and left ventricle (LV) as well as the left anterior descending artery (LAD) with its diagonal branch (D1) are visible. Pericardium (PC) is opened, allowing the dissection of the LAD, a silk snare with a counter bearing (asterisk) is placed around the artery (arrowhead, round magnification) and tightened for coronary occlusion. For more stable hemodynamic conditions the thorax is closed to a minimum. **(B)** Closed chest approach presenting an angiogram of a pig heart of the left coronary artery with LAD and left circumflex artery (LCx) in anterior-posterior view. Anatomical details of the coronary arteries can be identified. Placing a percutaneous transluminal coronary angioplasty (PTCA) balloon and inflating this leads to an occlusion downstream the LAD, here shown for a mid-LAD occlusion. RA, Right atrium; LA, Left atrium. Yellow area indicates area at risk during ligation/occlusion. Modified with permission after Sattler (7) and Sattler et al. (64).

A response similar to AMI caused by atherosclerosis or thromboembolic events in otherwise healthy hearts can be provoked by occluding a coronary artery in an experimental animal. This can be performed surgically by coronary vessel ligation in an open chest configuration (Figure 4A) or, less invasively, by catheter based techniques in closed-chest models including percutaneous balloon occlusion (Figure 4B) or microsphere injection (65, 66). Surgical ligation and balloon occlusion allow for ischemia/reperfusion models and correspond to transmural myocardial infarction seen as STEMI in patients (67). Microspheres induce local clotting in the heart microcirculation and lead to subsequent chronic myocardial ischemia (66). These techniques model AMI, the ultimate consequence of coronary artery disease. Less distinctive effects of coronary artery disease preceding AMI, including marginal effects of heart failure with chronic remodeling of autonomic innervation, inflammatory activation with its direct effect on the arrhythmic substrate (68), as well as development of collaterals are often missed.

The development of canine and porcine infarct models started with two-staged open chest models. A coronary artery was occluded to 50% of its diameter and occlusion was kept for 30 min before the artery was occluded completely and transmural AMI was induced (65, 69). These two-staged experiments often showed decreased arrhythmias and infarct size, most likely due to myocardial preconditioning (70). Two-staged models can be used to mimic the correlate of pre-infarction angina. Schwartz et al. moved from modeling the “healthy” human heart with first AMI toward acute-on-chronic myocardial infarction. In a first procedure, myocardial infarction by occluding the LAD was induced. Over a 2- to 4-week period, the animals developed anatomical and structural substrates, similar to those observed in clinical chronic artery disease, due to the chronic infarction in the LAD area. In a second procedure AMI was induced by LCx occlusion and the arrhythmic effects could be investigated (71).

While open chest models have been crucial in understanding AMI-induced arrhythmias, they come with several limitations: thoracotomy and subsequent opening of the pericardium influences thoracic pressure, cardiac filling and blood pressure and the surgery itself can lead to global ischemic preconditioning. In contrast, when percutaneous angioplasty is performed, the thorax and pericardium can be left intact, allowing for more stable hemodynamic conditions (72, 73). Further, anatomical limitations in choice of coronary occlusion site, such as vessels on the posterior side of the heart or intramural vessels, can be overcome.

### Infarct Location, Size, and Collaterals

Infarct location and occlusion sites within the coronary arteries can alter the incidence of ectopic beats and ventricular arrhythmias. Variation of occlusion site in the LAD in a porcine model (30) and anatomical variations of the LCx in an ovine model resulted in varying infarcted areas and different incidences of VF (31).

Since infarct size is dependent on the individual anatomical variation of coronary vessels and on the presence of collaterals, an important aspect when aiming for reproducible infarct sizes is the presence and degree of collateral flow. Humans have a well-developed network of coronary collaterals (74) that can be recruited rapidly during acute ischemia when direct flow is interrupted (75). Although only 20 to 25% of humans with healthy coronary arteries have sufficient collateral flow to prevent signs of myocardial ischemia during short vascular occlusions (76), those with coronary artery disease or prior myocardial infarction have a collateral flow that can be sufficient to prevent myocardial ischemia during coronary occlusion (77). In both humans and pigs it has been found that the degree of coronary flow reduction has direct influence on ventricular electrical and mechanical parameters (74, 78). Among animal models there is a large diversity in collateral perfusion: Non-diseased pigs, rabbits, and ferrets have low collateral flow. Dogs, cats, and guinea pigs, on the contrary, have a high collateral flow (75, 79).

Differences between human and animal exist in evolution of myocardial infarction within the myocardium at risk. Humans experiencing AMI show significantly slower infarct progression than animals (pigs, rats and dogs) with induced ischemia (80).

### Species Differences in Large Animal Models of AMI

Several different large animal models have been used over the last few decades in the setting of AMI and VF, during which some have become more popular than others. A PubMed search in May 2019 on studies conducted in dogs, pigs, sheep or primates found 448, 77, 9, and 7 publications, respectively [MeSH search terms used: “myocardial infarction” AND “ventricular fibrillation” AND (“dogs” OR “swine” OR “sheep” OR “primates NOT hominidae”)].

#### Dog

Dog models have provided deep insights into the complex electrophysiological processes and arrhythmias during AMI (32). The cardiac electrophysiology of the dog quite closely resembles that of man. Its anatomy and distribution of Purkinje fibers is similar to humans, providing a similar sequence of cardiac activation and ventricular depolarization, as can be observed in the ECG (54). However, ventricular repolarization shows marked differences in dogs, with much greater variability and inhomogeneity of repolarization across the ventricular wall (55, 81). The dog heart has been found to possess a rich collateral circulation (82), in contrast to pigs and humans, with the exception of patients with coronary artery disease. Recently, the use of dogs has been restricted by animal welfare laws, accompanied by rising expenses and the publications including dogs in the field have consequently declined markedly.

#### Pig

Pigs are widely used since their cardiovascular system in general, and the anatomy of the heart in particular, resembles that of the human. Despite the similarities, Landrace pigs with similar size to humans are only adolescents; full-grown adults weigh several 100 kg. In contrast to Landrace pigs, minipigs show slower growth rates accounting for a more stable heart/body weight ratio (83) and providing a more cost-effective option, especially in long term experiments (84). Cardiac similarity of pig to man is evident in many aspects, such as structure, electrical properties, and metabolic response to AMI (85). Since collateral flow is largely absent in pigs, a close modeling of AMI with a more controlled and solid infarct zone is possible (75). There are differences in electrical properties, Purkinje fibers and propagation speed of ventricular excitation (86). Pigs have a higher number of Purkinje fibers, spreading almost completely from endo- to epicardium, which is different to the predominantly endocardial presence in humans. Purkinje fibers have a 5–10 times higher conduction velocity compared to humans, leading to narrower QRS complexes when compared to humans (87). This results in a different orientation in excitation of the ventricles (87), which may impact the onset and maintenance of ventricular arrhythmias, especially during later phases of VF (88). In addition, when compared to humans, pigs show higher VF thresholds upon electrical stimulation (89).

#### Sheep

Hearts of sheep have fairly similar anatomical characteristics and physiological function as those of man. Their coronary circulation, as well as the anatomy of their conduction system is comparable to that of humans making sheep an interesting model for human AMI (90–92). However, only a few studies addressing AMI have been performed on sheep.

#### Non-human Primates

Whereas some earlier studies tried to exploit the close genetic similarity of non-human primates to man by using rhesus monkeys and baboons in AMI research (93, 94), today the use of monkeys is very limited. This is mostly due to logistical, ethical and monetary difficulties. LAD occlusion in unanaesthetized rhesus monkeys provoked cardiovascular response similar to man. VF was seen in 3/9 individuals (94). Additionally, despite close anatomical resemblance to humans, the hearts of these monkey species are small and have much faster sinus rhythm than man, making them less favorable in respect to electrophysiological resemblance (54).

## Pharmacological Targets

Despite survival rate being markedly decreased in AMI patients experiencing VF before or during primary PCI (8, 10) the prophylactic use of antiarrhythmic drugs is not recommended in the latest ACC/AHA guideline on STEMI therapy from 2013 in lack of effective therapy options (95). Only initiation of beta blocker therapy within 24 h after AMI onset is recommended as it has beneficial effects on VT/VF occurrence (96). However, AMI leads to a variety of pro-arrhythmic changes that can potentially pose pharmacological targets.

In the following section, amiodarone, save and effective in arrhythmia protection in the setting of AMI, and new drugs or experimental compounds in the setting of AMI induced VF that have been tested in large animal models or humans are reviewed. Drugs that have been proven harmful to human patients, like the class Ic antiarrhythmic drugs encainide and flecainide (97) or the class III drug d-sotalol (98) are not discussed here. An overview is given in Table 1. It is noteworthy that nearly all tested drugs were applied either before AMI was induced or during VF, while almost no experiment or clinical studies tested drug effects immediately *after* AMI but before coronary blood flow could be re-established. The temporal relationship between drug and ischemia can be crucial when aiming for translatability as the drug in human patients will not at all, or in lower concentrations, be present in the infarcted myocardium. The importance of this temporal relationship was shown for the class 1b agent aprindine were pre-treatment with the drug resulted in higher rates of VF during AMI than treatment applied after onset of AMI (117).

**Table 1:** Studies conducted on pharmacological targets in first acute myocardial infarction to prevent ventricular fibrillation including species and temporal relationship between drug treatment and ischemia.

**Drug/Target**	**Number of animals, species and study**	**Treatment before AMI**	**Treatment < 1 h after AMI**	**Treatment > 1 h after AMI**	**Treatment during VF**	**Outcome**
Amiodarone	60 dogs (99)				X	Amiodarone together with adrenaline and lidocaine improved defibrillation success rate compared to adrenaline and lidocaine alone
	114 sheep (100)	X				Amiodarone together with lidocaine decreased lethal arrhythmia
	18 dogs (101)			X		Amiodarone suppressed ventricular arrhythmia (given 24 h after AMI)
	24 dogs (102)			X		Amiodarone suppressed ventricular premature beats and VT (given 24 h after AMI)
	24 dogs (103)		X			Amiodarone prolonged AP duration and decreased dispersion. 10 mg/kg decreased vulnerability to rapid ventricle stimulation, while 20 mg/kg increased it
	18 pigs (64)		X			Amiodarone prevented VF, given 10 min after AMI onset
	3,026 humans (104)				X	No difference on 30 day mortality for amiodarone, lidocaine, or placebo
NHE1 blockage	16 dogs (105)	X				HOE642 reduced VF incidence, the occurrence of premature beats or VT was unchanged.
	19 pigs (106)	X				Continuous infusion of HOE642 before and during AMI reduced VF incidence from 9/11 pigs to 0/8 pigs.
	13 pigs (107)	X				Cariporide removed AP shortening during reperfusion, 10 min after AMI
K_ATP_ channel blockers	14 pigs (108)	X				HMR1893 attenuated AP shortening during AMI and reperfusion and improved excitation propagation during AMI
	15 dogs (109)	X				5-hydroxydecanoate attenuated AP shortening during AMI and reperfusion and improved excitation propagation during AMI
	68 pigs (110)	X				Thiazolidinedione drugs attenuated AP shortening during AMI. The treatment with rosiglitazone or HMR-1098 resulted shorter median time to VF (29 vs. 6 min)
	121 pigs (111)	X				Thiazolidinedione drugs shortened time to VF, reduced defibrillation success rate, attenuated conduction slowing and shifted ECG power spectra during VF to higher frequencies. The same effects were seen with glyburide but not 5- hydroxydecanoate
Gap-junction modifiers	62 dogs (112)			X		Rotigaptide increased gap junctional conductance and prevented induction of VT
	20 pigs (113)				X	Rotigaptide decreased defibrillation threshold and fibrillation amplitude in electrically induced VF. Return of circulation after defibrillation was not improved
I_f_ current blocker	54 pigs (114)	X				Ivabradine reduced heart rate, reduced AP shortening and increased VF threshold
	80 pigs (115)	X				Ivabradine delayed the time to onset of ischemia-induced VF
	22 pigs (116)	X				Repetitive episodes of 1-min ischemia were used. Ivabradine increased regional myocardial blood flow

*AMI, acute myocardial infarction; VF, ventricular fibrillation; VT, ventricular tachycardia; AP, action potential; NHE1, sodium-proton-exchanger of subtype 1; K_ATP_, Adenosine triphosphate-sensitive potassium channel; I_f_, funny current*.

### Amiodarone

Amiodarone is believed to be one of the most effective antiarrhythmic drugs for the treatment of a variety of different arrhythmias. Effects of amiodarone differ between acute and chronic treatment. In acute treatment the drug inhibits inward Na^+^ and Ca^2+^ currents and outward K^+^ currents (I_Kr_, I_Ks_, I_K,Ach_, and I_K,Na_) (118) and has non-competitive beta blocking effects (119). Acute intravenous applications mainly lead to slowing in atrioventricular conduction and have only mild effects on the effective refractory period and little effect on QTc intervals, whereas chronic treatment slows conduction in almost all cardiac tissues and prolongs QTc interval (118).

Treatment with amiodarone has been tested in a variety of animal models before and after AMI, as well as during VF. Overall, animal models showed reduced ventricular arrhythmia (100–102), decreased dispersion (103) and improved defibrillation success rate (99). We showed reduced VF incidence rates with amiodarone when given 10 min after AMI onset (64). In humans, little evidence is available for the prophylactic use of amiodarone in the acute setting of AMI since randomized trials mainly looked at unselected OHCA cohorts (120, 121). As reported in a multicenter study on 3,026 patients, survival in OHCA patients was not improved by the use of amiodarone or lidocaine (a Na^+^ channel blocker) when administered during VF (104). Yet data from a retrospective analysis focusing on VT and VF during AMI suggest a positive effect of treatment with amiodarone or lidocaine on survival (122). No randomized trials in humans, investigating prophylactic treatment with amiodarone after onset of AMI to prevent VT/VF in the early phase, exist.

### NHE1 Blockage

The Na^+^/H^+^ exchanger of subtype 1 (NHE1) is an important proton-extruding mechanism in cells including cardiomyocytes (123). During AMI, intracellular pH decreases due to anaerobic metabolism, leading to an activation of NHE1 (124). Exchange of the excessive proton load for Na^+^ causes intracellular Na^+^ accumulation that could indirectly affect the activity of other ion transporters, particularly the NCX, which may lead to ventricular arrhythmogenicity.

Pharmacological NHE1 inhibition in AMI animal models has only assessed pre-treatment (105–107). These reports showed reduced VF incidence (105, 106) without changes in other ventricular arrhythmias (105), and eliminated reperfusion induced AP shortening (107). In humans, NHE1 inhibition during AMI has not been assessed.

### K_ATP_ Channel Blockers

The ATP-sensitive K^+^ (K_ATP_) channels generate an inwardly rectifying outward current that is activated when the intracellular ATP concentration is low, such as during AMI. K_ATP_ activation during myocardial ischemia is partly responsible for the abbreviation of the ventricular AP and protects the heart from damage during ischemia by preventing Ca^2+^ build-up (125). K_ATP_ channels may represent an intriguing inhibitory target for antiarrhythmic therapy by reducing AMI-induced AP shortening. In several large animal studies, inhibition of K_ATP_ suppressed AMI-induced AP shortening (108–110) and improved excitation propagation (108, 109), however, time to VF was shortened (110, 111). This suggests that the protective effect of preventing intracellular Ca^2+^ build-up outweighs any possible anti-arrhythmic effect of reducing AP shortening.

### Gap-Junction Modifiers

Cardiac gap-junctions, with connexin 43 (Cx43) as their main isoform, provide communication and connections between cells, allow the exchange of small molecules, and play an important role in the propagation of electrical excitation in the heart (126). Gap junction inhibition during AMI reduces conduction velocity and increases defibrillation thresholds (127). In contrast, gap junction modifiers, such as rotigaptide, increase conduction velocity by increasing functional coupling of gap junctions at the intercalated disc (128). Rotigaptide prevented programmed stimulation-induced reentrant VT during MI (112) and decreased defibrillation threshold during prolonged VF (113). This, however, did not translate to improved rates of return of spontaneous circulation (113). Mitochondrial Cx43 is involved in the energy metabolism in the heart (129) and Ca^2+^ handling (130). Affecting these processes could produce unwanted side effects.

### I_f_ Current Blockers

Sinus node tissue shows spontaneous depolarization by a complex interaction of multiple inwardly and outwardly directed ion currents, including the funny current (I_f_). This current is carried primarily by the hyperpolarization-activated cyclic nucleotide-gated (HCN4) channels primarily conducting Na^+^. In the setting of AMI, extra-nodal I_f_ in the left ventricle can be increased leading to proarrhythmic tendencies (131).

Ivabradine selectively blocks the I_f_ current in a use-dependent manner, resulting in reduced heart rate without negatively affecting hemodynamics (132). Ivabradine pre-treatment in AMI reduced heart rate, increased VF threshold and prevented AP shortening in the ischemic area without changing myocardial contractility (114). Ivabradine also delayed the time to ischemia-induced VF by preserving myocardial metabolic energy status (115) and lowering heart rate (116).

### Autonomic Modulation

Besides pharmacological interventions on ion channels, techniques to modulate autonomic dysfunction in AMI by increasing vagal activity and reducing sympathetic hyperactivation are arising (133). The different techniques have been reviewed recently by Lai et al. (134). Briefly, vagal activity can for example be increased by the tragus nerve, carotid baroreceptor and vagus nerve stimulation, sympathetic activity can for example be decreased by spinal cord stimulation, stellate ganglion denervation or neuromodulation and renal sympathetic denervation.

Modulation of the sympathetic nervous system by renal denervation reduces ventricular ectopy and VF in a porcine model of AMI. During reperfusion AP shortening and spontaneous arrhythmias were not affected by this modulation of the autonomic nervous system (135). Direct stimulation of cervical pre-ganglionic parasympathetic fibers activates overall cardiac vagal tone (136). Vagal stimulation, performed shortly after the onset of an acute ischemic episode in conscious animals with a healed MI, can effectively prevent VF independent of heart rate reduction (137). Spinal cord stimulation also has an antiarrhythmic effect on spontaneous non-sustained VT and sustained VT during ischemia-reperfusion in association with a reduction of repolarization alterations independent of any effect on infarct size (138).

## Summary and Conclusion

AMI complicated by VF is one of the most common causes of SCD while treatment options still remain limited. Even if SCD is averted, VF in AMI patients is associated with decreased survival rates. Several risk factors like sex, family history, life style factors and alcohol consumption are known from epidemiological studies. As studying AMI-induced VF is difficult in humans, large animal models provide an important translational approach to study AMI complicated by VF.

A variety of large animal models resembles human cardiac physiology and can be used to investigate mechanisms of risk factors or new treatment modalities. There are several important factors to take into account when studying spontaneous VF during AMI: the animal with its specific cardiac anatomy and electrophysiology; the anesthesia and its effect on heart rate and autonomic regulation; and the method, size and location of coronary occlusion and the resulting ischemic area.

Although a number of promising pharmacological treatment regimens such as K_ATP_ blockers, gap-junction modifiers, I_f_ channel blockers, and NHE1 blockers have been investigated in large animal models, the applied experimental design is often insufficient to be successfully translated into clinical trials. Many of the experiments evaluating new antiarrhythmic drugs are performed pre-treatment before AMI onset, thereby results in drug accumulation also in the infarcted area. This can both lead to electrical and metabolic stabilization or destabilization of the infarcted area. For a successful translation into clinical studies it is of importance to adjust the experimental design according to human situation.

## Author Contributions

SS and LS: concept, design, drafting article, critical revision of article, and approval of article. DL, AL, JT-H, and TJ: drafting article, critical revision of article, and approval of article. AL: critical revision of article and approval of article.

### Conflict of Interest

The authors declare that the research was conducted in the absence of any commercial or financial relationships that could be construed as a potential conflict of interest.
